# Learning science with YouTube videos and the impacts of Covid-19

**DOI:** 10.1186/s43031-022-00051-4

**Published:** 2022-04-11

**Authors:** Wayne Breslyn, Amy E. Green

**Affiliations:** College of Education, University of Maryland, College Park, USA

**Keywords:** Instructional video, Learning ecosystems, Online learning

## Abstract

This study investigates student and teacher use of online instructional YouTube chemistry videos in the context of the Covid-19 pandemic. Data were collected from a global sample of students (*n* = 1147) subscribed to the first author’s popular chemistry education YouTube channel. Participants were in secondary school or college and reported having learned science in a variety of contexts including completely online, blended, or completely in-person. The data collection instrument, an online questionnaire, was designed to detect both quantitative and qualitative changes in the use of instructional video. In addition, statistics for the overall YouTube chemistry education channel for 2018 through 2021 were compiled to provide evidence of video viewing trends with a large sample (98.6 million video views) over a timeframe encompassing before and during the Covid-19 pandemic. Findings indicate that students’ personal use of video for learning science increased substantially during the pandemic. However, for the majority of teachers, the use of video to support online learning during the pandemic either remained the same or declined. Post-pandemic, students plan to continue using science videos for learning and want teachers to do the same.

## Introduction

The COVID-19 pandemic has had profound impacts on teaching and learning around the world, significantly altering instructional norms for students at all levels. The disruption of in-person learning in schools and universities greatly impacted student access to traditional instructional resources and accelerated changes in the modes of delivering education (United Nations 2020). Recent research shows that learning continuity during the height of the pandemic was dependent upon not only the availability of online communication technologies and resources, but also on the augmented capacities for students to learn independently (Reimers, 2022). At the time of conducting and reporting our research for this special issue, much of the world is still in the midst of the Covid-19 pandemic. While many schools have reopened, the impact of the time spent in virtual learning environments on future learning remains uncertain.

Even with virtual support from teachers, the shift to online learning has heightened the demands on students’ abilities to use web-based technologies to support and monitor their learning. This was evident from the rapid and sustained use of the first author’s chemistry instruction YouTube channel during the onset of the pandemic and provided the impetus for the current study as well as access to study participants.

Demands on teachers to leverage these technologies to support curriculum-based instruction have also increased. This study represents an effort to capture the impact that the COVID-19 pandemic has had on students’ habits and preferences for using instructional science videos before and during periods of virtual learning as well as students’ recommendations for how these resources should be used as schools return to traditional in-person learning methods.

The YouTube video-sharing platform resource was chosen as a research sample due to its expanding popularity as one of the most widely used social media sites among young Americans, with 95% of 18–29-year-olds reporting having used it in 2021 (Auxier & Anderson, 2021).

Globally the platform is widely used with 2.1 billion users in 2021 and a projected 2.8 billion users by 2025 (Degenhard, 2021).

Our emphasis on video as a major component of the constellation of resources students use to learn science is guided by the learning ecology theoretical framework (Barron, 2004, 2006, 2016; Corin et al., 2017; Falk et al., 2016; Jackson, 2013; Staus et al., 2021; Traphagen & Traill, 2014). This theoretical perspective conceptualizes and explores science learning within educational *ecosystems* made up of relationships between the physical, virtual, and in- and out-of-school environments as well as the associated resources, artifacts, and processes through which people learn. In this study, we explore the use of video in different contexts relative to other components of the education ecosystem such as textbooks, lectures, and laboratory work.

Our ultimate aim for this research is to document how students have adapted to learning during the disruption caused by the pandemic (in the context of an extensively used online resource) and, looking forward, to describe how students anticipate the changes and resulting adaptations will impact science learning in the future. We anticipate video will continue to maintain a prominent role in how students approach their own personal science learning, but the effects that the pandemic-driven shift to online learning will have on formal approaches to in-person instruction in the future is unknown. Our research project lays the groundwork for understanding student use of video and defining questions to guide future research around this growing resource that students are using to learn science in formal and informal contexts.

## Background and theoretical framework

### Learning ecosystems

Our exploration of online instructional videos as resources for learning science is guided by the ecological perspectives of the learning ecosystems theoretical framework (Barron, 2004, 2006; Corin et al., 2017; Falk et al., 2016; Jackson, 2013; Staus et al., 2021; Traphagen & Traill, 2014). Ecologists studying natural ecosystems explore the interconnected relationships between biotic and abiotic components in physical environments and the cycles of matter and energy that connect them. The learning ecosystems theoretical framework applies the metaphor of an “ecosystem” to conceptualize and study learning. Learning ecosystems are framed as “the complex of living organisms in a learning environment (e.g., students, educators, resources), and all their interrelationships in a particular unit of space” (Giannakos, et al., 2016, p.106). Students and teachers are often considered the primary, interrelated ‘actors’ that both shape and are influenced by the physical and virtual spaces in which learning happens as well as the resources used. These learning spaces include not only traditional classroom-based schooling, which typically features resources such as slides, lectures, peer discussion, laboratory tools, and books; but also informal spaces such as zoos, museums, and parks. Social media and other digital communication technologies such as television, podcasts, webinars, email, instant messaging, online document sharing, and online videos are also increasingly becoming part of the ecosystem of science learning.

The learning ecosystems framework allows curriculum designers, practitioners, and, in our case, researchers to examine the individual pieces of the ecosystem and how they connect in order to achieve a holistic understanding of learning. It enables us to see the integrations of experiences synergistically across settings and time rather than attending to single moments of learning (Bell et al., 2009; Corin et al., 2017).

Learning ecosystem models typically situate students at the center of a “system” (Corin et al., 2017) and attend to interactions and relationships between the learner and *sources* of learning resources and/or situations in which these resources are accessed (e.g., families, after-school programs, museums, or classrooms). Current models generally do not identify or define in depth the *types* of resources (e.g., videos, textbooks, websites, and discussion) that students are using to learn science, nor do they describe the *relationships* between when, how, and why these resources are being used in academic programs (e.g., Hecht & Crowley, 2020; NRC, 2015; United States Department of Education, 2015). We apply the learning ecosystems model to explore and better understand these individual components and their relationships from the student perspective. In this way, understanding the interactive parts of the system can help us construct a more meaningful understanding of the whole.

**Fig. 1:**
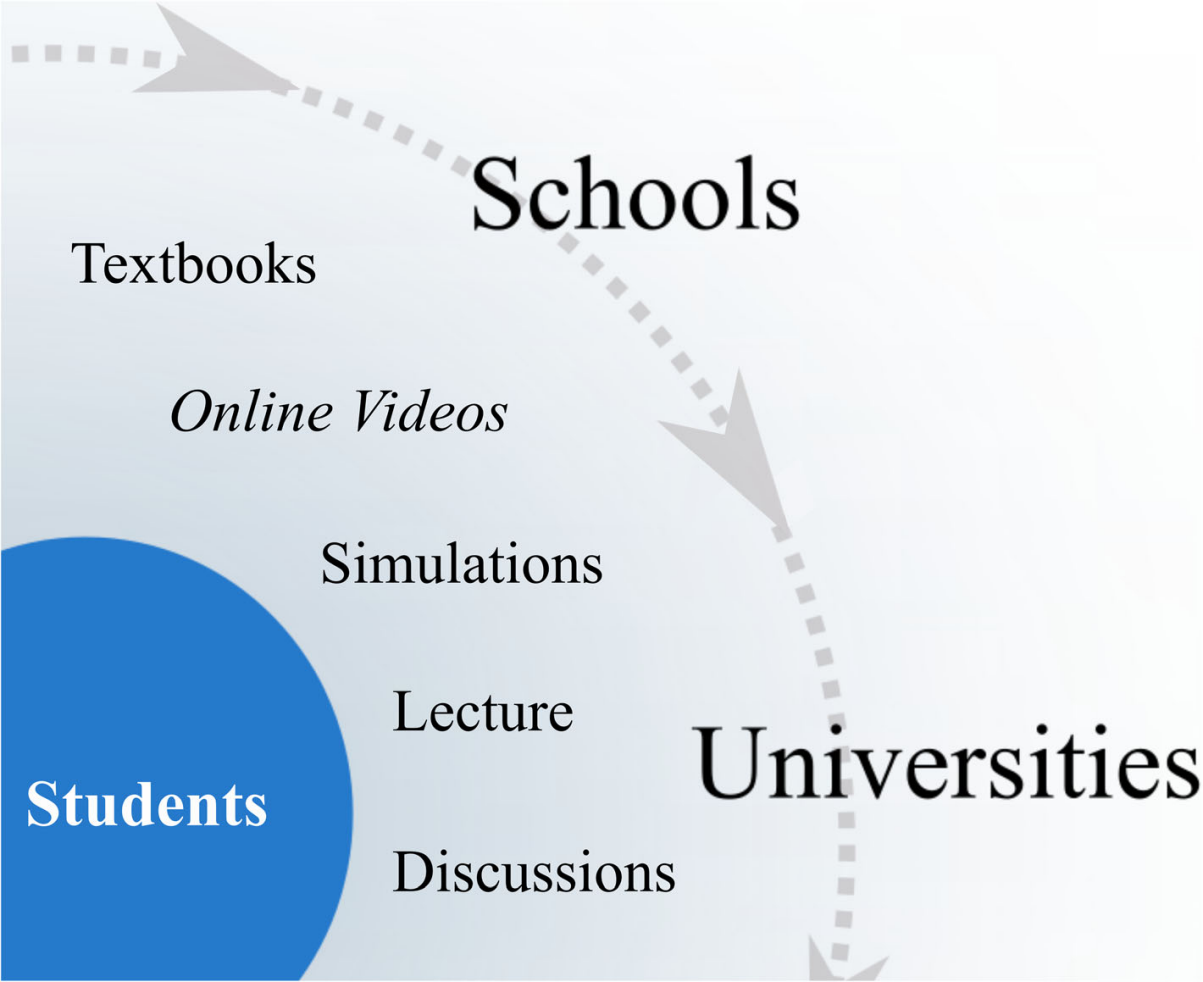
Example of the parts of learning ecosystem explored in this study

Our research specifically acknowledges that decision-making within learning ecosystems is driven by teachers and students in different contexts and for different purposes and represents an effort to provide more insight into how students are using videos as resources within science learning ecosystems. As shown in Fig. 1, we focus specifically on students’ use of video relative to other resources for academic learning and use the learning ecosystems theoretical perspective to better understand its role—in other words how, when, and why students are utilizing video relative to other resources to support their learning. For example, we explore when, how, and why videos are selected by students for self-driven activity versus when they are being used at the direction of the teacher to support school-based activities. We further leverage the learning ecosystems framework to understand student preferences for teacher-driven decision-making in post-pandemic classroom learning.

### Online instructional YouTube videos as a learning resource

Research over the past decades indicates that science learning is a cumulative process and that experiences both in and outside of school contribute to how we come to understand science (Bell et al., 2009; National Research Council, 2010). With online media consumption growing (now greater than the consumption of traditional media) for young people (Westcott et al., 2022), the potential role of video has grown and will continue to grow, meriting an opportunity for scholarly exploration into connections between instructional science videos and learning. The pandemic-driven shift to online instruction has accelerated the frequency with which many students are using YouTube videos to support science learning; however, video continues to be underrecognized by scholars and practitioners as serving productive roles within science learning ecosystems. Students are using videos, but we are uncertain about when, for what purposes, and how they are using them in relation to other resources (e.g., textbooks, lectures). These understandings are essential for informing post-pandemic curricular and pedagogical decisions that will impact students, many of whom spent a year or more using online technologies to support and monitor their learning.

Since the inception of video-sharing services, the use of science videos for classroom instruction and individual learning has consistently grown (Hibbert, 2014). Our research specifically studies online instructional science videos. These videos have more academic-based content, relating directly to science taught in schools, and tend to be overviews of a science topic and include step-by-step explanations and solutions to problems. They most often explain concepts in a lecture format, frequently using a digital whiteboard. Many involve visualizations and simulations of science phenomena. Instructional science videos on the YouTube platform are created by a variety of individuals. The vast majority are produced by individual science educators and/or educational organizations such as Khan Academy.

We recognize that the role of YouTube in education is a nascent field of study and provide a detailed background on the contextual factors surrounding the online video sharing platform. In Table 1 we also provide descriptions of terms commonly used on the YouTube platform.

**Table 1:** Key Terms for the YouTube Video Sharing Platform

• *YouTube*: An online video sharing and social media platform where users can view and post videos. • *Channel*: An individual’s own personal presence on YouTube.com where they can post, organize, and engage in discussions about the videos they create and upload. • *Subscriber*: An individual who chooses to receive updates when videos are posted to a specific channel. • *Community Tab*: Section of a YouTube channel for announcements, polls, and updates. • *Poll*: An option on the Community Tab for feedback from subscribers. • *Notification*: A message sent to subscribers via the YouTube platform when a video is posted. • *Endscreen*: An image of a video linking to another video or website shown at the end of a video.	

Students cite a variety of reasons for their use of YouTube videos for learning and many of these reasons are related to the agency that students have for controlling the instructional delivery. For example, many highlight the ability to pause, rewind, or forward as allowing them to work at their own pace and focus specifically on where they need support (Stockwell et al., 2015). Students also report appreciating the value of YouTube for allowing them to essentially select the teacher from whom they receive the instruction. Selection factors include tone of voice and affect in delivery. Furthermore, in comparison to traditional instructional resources like textbooks, students see YouTube videos as being more personal, with 59% of students aged 14–23 preferring YouTube videos over textbooks (Harris Poll, 2018).

From a research perspective, online instructional videos have been shown to be productive for supporting learning for many diverse learning demographic groups. For example, students learning science in school in a second language (e.g. ELL students) find value in that they can access automatically generated subtitles for most major languages. This, coupled with pause and rewind, supports learning with added benefits of improving English listening, writing, and speaking skills (Warschauer & Grimes, 2007). Videos also offer opportunities for students to learn from content experts from diverse racial, ethnic, and linguistic backgrounds.

In addition, nearly one in five US students attend rural schools, and although typically less diverse, more than one in four is a student of color (School Superintendents Association, 2017). Rural schools often struggle to fill positions (Tran & Smith, 2020) and are more likely to employ teachers without graduate degrees and/or extensive college-level science coursework than those in urban districts (Echazarra & Radinger, 2019). Further, rural schools typically have fewer options or opportunities for students to take advanced science classes as part of a formal academic program (Gemin et al., 2018). For these students, educational videos can serve to supplement instruction. Videos also offer opportunities for students to learn from content experts from diverse backgrounds and teaching styles.

Finally, students with learning disabilities make up 14% of the student population in the US (NCES, 2020). Research has shown that student performance across multiple measures, including problem-solving accuracy and independence, may be improved with video (Ayres & Langone, 2005; Satsangi et al., 2021). These students benefit from the ability to slow down text, change font size and color, and enable subtitles as well as receive instruction in a variety of modalities.

### YouTube analytics analysis

Video content creators on YouTube have access to extensive analytics on how viewers interact with their channel and with video content. YouTube Analytics, the reporting platform for YouTube creators, offers the ability to generate comparisons of video views over date ranges. While this data cannot be connected to any individual viewer, the overall information available can be a valuable tool for understanding how, when, and who is viewing video content.

Within the current study, this functionality is used to compare video views from the first author’s YouTube channel over four years, 2018–2021. This dataset is large, with 96.8 *million* video views from 1/1/18 to the present (10/10/21). The analysis provides a broad view of when videos were viewed and can graphically display volume across different years showing the frequency with which instructional chemistry videos on the first author’s channel were viewed before and throughout the pandemic.

**Fig. 2:**
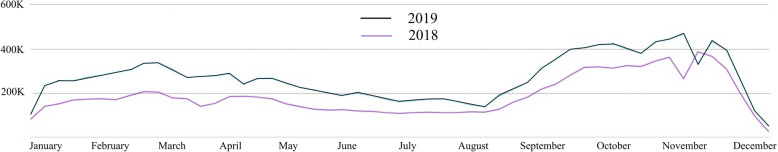
Comparison of video views for 2018 (purple) and 2019 (black)

A comparison of videos viewed on the first author’s YouTube channel during the 2018 and 2019 calendar years provides a pre-pandemic baseline of video use by students (Fig. 2). The number of views follows the same pattern in both 2018 and 2019 with July and August being lower due to summer vacation for U.S. students. This is followed by a steep increase starting in September, a dip due to a decrease in U.S. viewers during the Thanksgiving break in the U.S., and then a steep decline heading into the new year. Due to the addition of content over the previous year, and during 2019, views for 2019 are consistently higher.

**Fig. 3:**
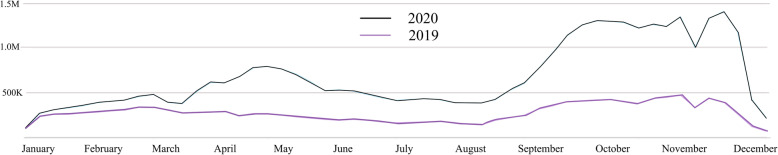
Comparison of video views for 2020 (black) and 2019 (purple)

Looking at a comparison of 2019, a more typical year, to 2020, highlights changes in the number of videos viewed (Fig. 3 below) before and during the pandemic. Note that the scale has changed in Fig. 3 with the upward bound now at 1,500,000, over double the scale in Fig. 2.

There is an expected growth in video views from the addition of new content for January and February. However, in March of 2020 views decreased and then rapidly increased. This coincides with the Covid-19 pandemic reaching global dimensions and affecting a large number of countries. The elevated levels continued throughout the year with a steep increase in September through December of 2020.

**Fig. 4:**
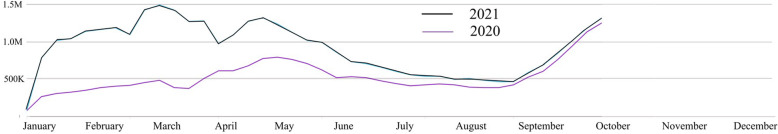
Comparison of video views for 2021 (black) and 2020 (purple)

Figure 4 shows a comparison of 2020 and 2021. Views continue to increase in 2021 until June when they begin to more closely follow 2020. By September 2021 there is only a small increase in views for the year, however this is still more than *double* pre-pandemic levels, even with many students returning to in-person learning. This suggests that the growth in student use of videos may be a lasting change.

While these analytics are valuable for providing insight into viewership and allow us to make inferences to predict future video use, there are several limitations to the data. First, it represents only one YouTube channel, and other channels may differ. When data presented in Figs. 2, 3, and 4 were compared to a separate chemistry education YouTube channel, both data sets showed the same trends across the time frames presented (M. Maribel, personal communication, October 25, 2021). However, a comparison accoss multiple YouTube channels was not possible.

Second, additional content added to the channel over the timeframe, growth in competition from other similar channels, as well as changes in how search algorithms promote content, could also lead to changes seen in year-to-year comparisons. Complete analysis considering the addition of content, changes in YouTube and search ranking algorithms, and growth of YouTube viewership overall are outside the scope of this paper. However, the 2018 and 2019 comparison provides a baseline that partially controls for these factors.

### Research questions

Learning ecosystems are dynamic and their characteristics are variable. A disruption to any component within the system can lead to shifts within individuals and populations of individuals. From an ecological perspective, it is reasonable to assume that the pandemic has, at least temporarily, impacted the relative stability of science learning ecosystems as instruction moved online and access to traditional learning spaces, processes, and physical resources became limited. Our research takes a closer look at the impacts these disruptions have had on the relationships between components of the science learning ecosystem with a specific examination of the impact the pandemic may have had on the role of YouTube videos for learning. We also investigate the impacts on the system as a whole to understand and possibly predict the resilience and recovery of the system moving forward.

Online analytics indicate that the number of video views across many education-focused channels increased during periods of online learning, however little is known about how, why, and by whom these additional views were being used. Our research focuses on data collected from a sample of self-identified learners from the first author’s YouTube science channel and video usage statistics across the channel. We investigated three primary research questions:

- *Has student use of instructional science video changed during the Covid-19 pandemic and, if so, how?*
- *Has teacher use of instructional science video changed during the Covid-19 pandemic and, if so, how?*
- *What are students’ views for the role of online video once in-person learning resumes?*

## Methods

### Study design

**Fig. 5:**
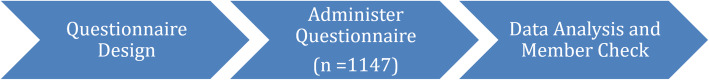
Research design

Our research design uses a mixed-methods approach to collect both quantitative and qualitative data (Fig. 5) on students’ reported use of video within science learning ecosystems before and during the pandemic (through summer 2021) as well as their views on the role of science videos once in-person learning resumes. The primary source of data was an online questionnaire. To extend and clarify findings after the initial questionnaire, a shorter follow-up questionnaire on language learning status as well as a poll to member-check findings were also administered. Data on the overall use of the first author’s YouTube channel was analyzed for 2018–2021 (presented in the Background and Theoretical Framework section) to triangulate findings on changes in the frequency of video viewing.

### Data collection and participants

Participants were students recruited from the first author’s YouTube chemistry education channel (*N* = 400,000). A total of 1307 completed the online questionnaire. Of these, 1147 were currently taking a science class or had taken a science class in the past year and were included in data analysis. Data were collected over two months from June 18th to August 18th, 2021.

The sample consists of participants from 83 countries. Table 2 lists countries that represent one or more percent of the total sample. These twelve countries represent 84% of the total sample. The percentages are rounded to the nearest percentage point.

**Table 2:** Countries with Highest Participation

United States: 30%	United Kingdom: 2%
India: 24%	Brazil: 2%
Canada: 5%	South Africa: 2%
Malaysia: 5%	Indonesia: 2%
Philippines: 4%	Nepal: 2%
Singapore: 4%	Pakistan: 1%
Australia: 4%	Bangladesh: 1%

*Note*: Percentages are rounded to the nearest percentage point

Participants represented a range of ages with 92% reporting their age between 13 and 24 years. Ages are summarized in Table 3.

**Table 3:** Ages of Participants

Under 13	13–17	18–24	25–34	34–44	45–54	55–64	Over 65
1%	62%	30%	4%	1%	1%	1%	0%

*Note*: Percentages are rounded to the nearest percentage point

Overall, 50% of participants were male, 46% female, 3% preferred not to answer, and 1% reported non-binary or other. The academic level is made up of primarily high school (62%) and undergraduate students (28%). Pre-high school students (7%) and graduate students (3%) are also represented in the data.

Responding to the question “How often can you view videos without problems?”, 42% stated they can always view videos, 52% often, 4% seldom, and 1% never. Participants reported accessing online video via a laptop or Chromebook (53%), desktop computer (24%), mobile device (18%), or tablet (5%).

**Fig. 6:**
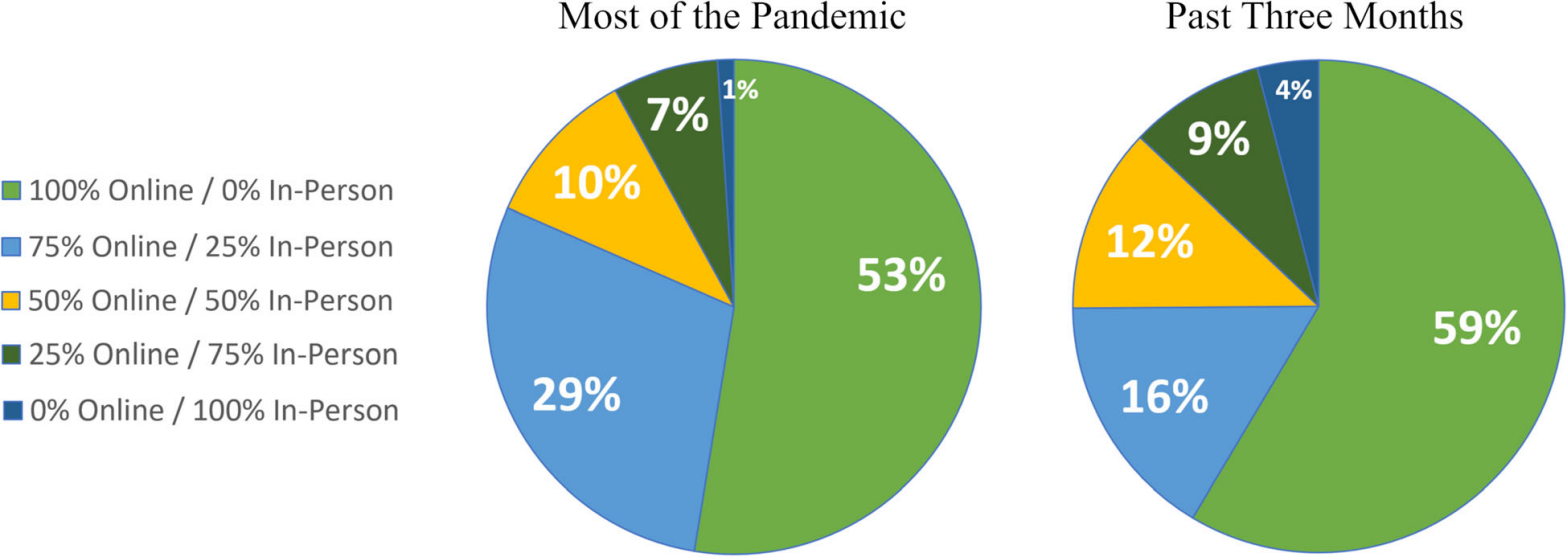
Learning contexts during the pandemic

The majority of data were collected from June 18th through July 18th (997 of the 1147 responses) with data collection continuing to August 18th, 2021. At the time of data collection, 59% of students reported being in 100% online instruction for the past three months as compared to 53% for most of the pandemic (Fig. 6). Only 4% reported 100% in-person for the past three months and only 1% for most of the pandemic. Note that in Fig. 6 the heading “Past Three Months” refects the date at the time the participant completed the questionnaire.

Data were not collected on whether English was a first or second language in the initial questionnaire. However, since many videos on YouTube are in English a follow-up was conducted one month later with a sample of 424 students from the same population. From this sample, 49% percent stated that English was a second language.

Due to the large and geographically distributed sample, participants under the age of 18 years of age participated in the questionnaire and poll anonymously and no identifying information was collected. Participants over 18 were given an option to leave an email address for further follow-up.

### Sample selection and recruitment

Participants for this study were recruited from an international population of subscribers to the first author’s YouTube chemistry education channel (*N* = 400,000). The channel features several minute-long instructional videos designed to help clarify complex chemistry concepts through worked examples and visualizations. Participants were contacted through the YouTube platform messaging system in accordance with IRB guidelines. Due to the community aspect of the YouTube channel involved, incentives for participation are unnecessary.

Per Institutional Review Board (IRB) guidance, all subjects participated in questionnaires and polls anonymously and no identifying information was collected. Participants indicated assent/consent directly on the online survey.

Note that many participants are subscribed to a variety of chemistry and other science YouTube channels. We anticipate that the sample in this study is representative of students studying chemistry through online videos, not just the first author’s channel.

### Data Collection & Analysis

#### Data collection instrument: online questionnaire

The data collection instrument was a questionnaire consisting of 19 items and was administered using Google Forms. In addition to demographic information, the form included ranked-choice, Likert scale, and open-ended items. An iterative design of the questionnaire was undertaken to strengthen the validity of the instrument in the context of participants of multiple ages and countries where English is often a second language.

To address issues of validity for this geographically and academically diverse group of participants we conducted multiple iterations of testing, analysis, and instrument revision by engaging groups of users reflective of the population in cognitive “walk-though” sessions in which each participant took the survey and provided real-time feedback to the researchers. Ten students and two teachers, including a high school science resource teacher who holds a Ph.D. in science education, participated in these think-aloud interviews using the instrument. After each interview, the questionnaire was revised. A Ph.D. expert in questionnaire design was then consulted, revisions were again made, and the form was then field-tested with a broader audience: 22 students from 10 countries.

Following analysis of the initial questionnaire, a second questionnaire was administered to determine the language learning status of participants.

#### Data analysis

The questionnaire collected both quantitative and qualitative data from each participant. Prior to analysis, data were checked for any duplicate submissions, missing data, or blank records. Data for all respondents were then sorted to focus exclusively on participants who were currently taking a science class or had taken a class in the past year. These records (*n* = 1147) were then analyzed. Quantitative data were summarized and described in text and in charts.

Analysis of qualitative data took place iteratively beginning with questionnaire data. An initial set of codes were developed from participants’ written text by the second author and documented in a codebook. Using these initial codes, a subset of data was coded by the first researcher.

During the process, as codes were added, modified, and expanded upon, the codebook was further developed including a description of the code, examples, and exclusion criteria.

Prior to coding the entire dataset, the second author coded a subset of data to assess Interrater Reliability (IRR). Initially, to ensure both researchers were familiar with the codebook and to provide further refinement, the first and second researchers collaboratively coded a subset of data from each source. As a result, modifications, primarily collapsing codes and adding clarifying descriptions for the inclusion and exclusion criteria, were made to the codebook based on discussions between researchers. After modifications were made to the codebook a different subset of data was independently coded by both researchers and IRR was calculated and found to be 94%.

## Findings

In our examination of the influence of the pandemic (through summer 2021) on the use of science online video within educational ecosystems, we focus and consider our findings in three areas: student reports of their *own* use of video before and during pandemic-driven virtual learning, student reports of *teacher* use of video before and during virtual learning, and students’ reporting of whether and how they think teachers should use video with students when classes return to primarily in-person learning.

### Student use of video before and during pandemic-driven virtual learning

#### General student use of video

Looking specifically at online video, we asked students to compare the frequency with which they used instructional science video before and during the pandemic. These data focus on student-directed use of video without guidance from their teachers. We asked:

*How did you use online SCIENCE instructional videos BEFORE the pandemic?*

*How did you use online SCIENCE instructional videos DURING the pandemic?*

**Fig. 7:**
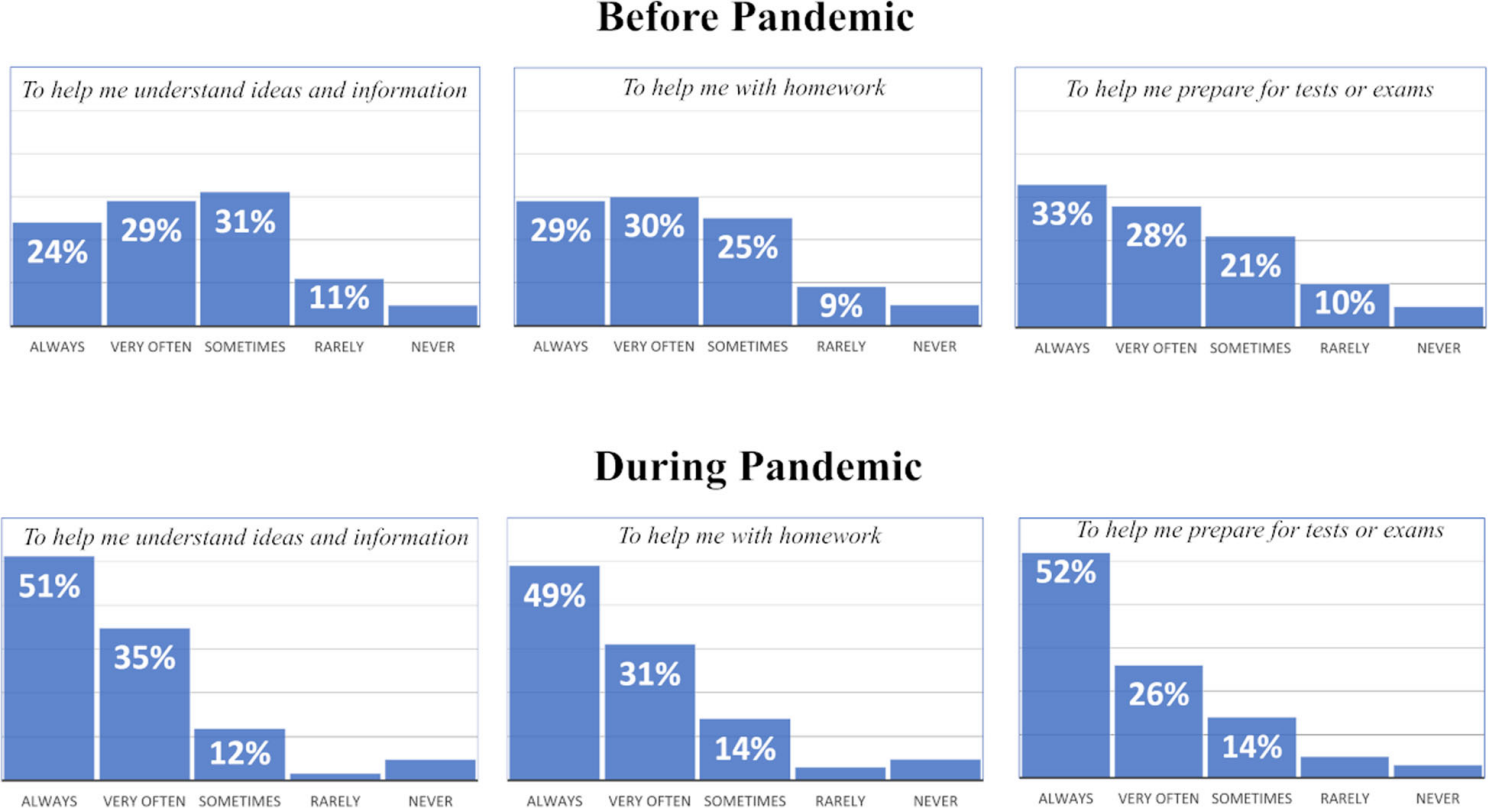
Student-driven use of video before and during the pandemic

Figure 7 shows students’ reported use of video to support three student-driven goals: *understanding ideas and information*, *homework*, and *preparing for tests and exams*.

In all three areas, students’ use of video increased during the pandemic. The percentage of students who used videos to help them understand ideas and information always or very often before the pandemic (53% collectively) and during the pandemic (86% collectively) indicated the most significant increase. These data raise many additional questions including which factors contributed to this increase (for example, increased accessibility of online videos in virtual environments and/or decreased access to teacher-based support), and whether students will continue to turn to video for learning support when in-person learning resumes.

### Student use of video compared to other resources within the learning ecosystem

**Fig. 8:**
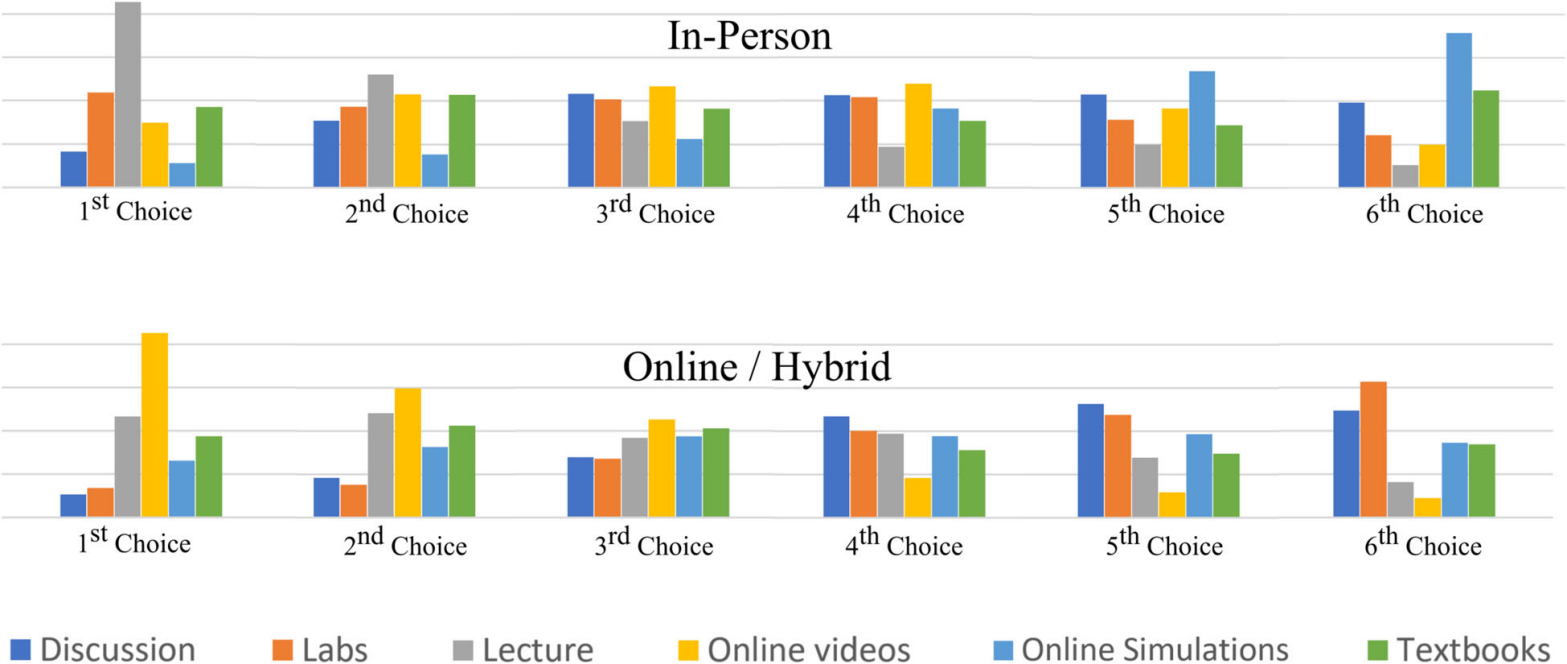
Students’ ranked choice for learning resources

Data in Fig. 7 reported changes in the *frequency* students used video for learning chemistry. In Fig. 8 below, data for the *type* of resources students used are presented for both in-person and online/hybrid learning contexts. In this sense, the data provides information on changes in students’ use of video relative to other learning resources in the chemistry learning ecosystem. We asked:

*In general, how effective are the following for learning SCIENCE IN-PERSON?*

*In general, how effective are the following for learning SCIENCE ONLINE and/or in HYBRID settings?*

Students were asked to rank resources for six different learning resources: Discussion, Lecture, Labs, Online Videos, Simulations/Visualizations, and Textbooks. Figure 8 presents data for students’ ranking of these resources in the contexts of In-Person learning and Online/Hybrid learning.

When learning in person, students report lecture to be their first choice (38%), followed by labs (20%), and then by textbooks (17%). Moving to an online/hybrid context, online videos become the first choice (39%), followed by lectures (20%), and then by textbooks (17%). In reviewing these data, our attention was drawn to the *consistency* in students’ preferences for using textbooks during in-person and online/hybrid learning contexts. This suggests that the context in which students are learning with textbooks does not have a meaningful impact on their preferences for using textbooks to accomplish academic tasks, in contrast to video.

### Teacher-driven use of video before and during pandemic-driven virtual learning

Our research also sought to better understand students’ perceptions of the frequency with which the use of video to support academic tasks was initiated by the teachers. We asked: *As part of an academic assignment* and for *an in-class activity*,

*How did you use online SCIENCE instructional videos BEFORE the pandemic?*

*How did you use online SCIENCE instructional videos DURING the pandemic?*

**Fig. 9:**
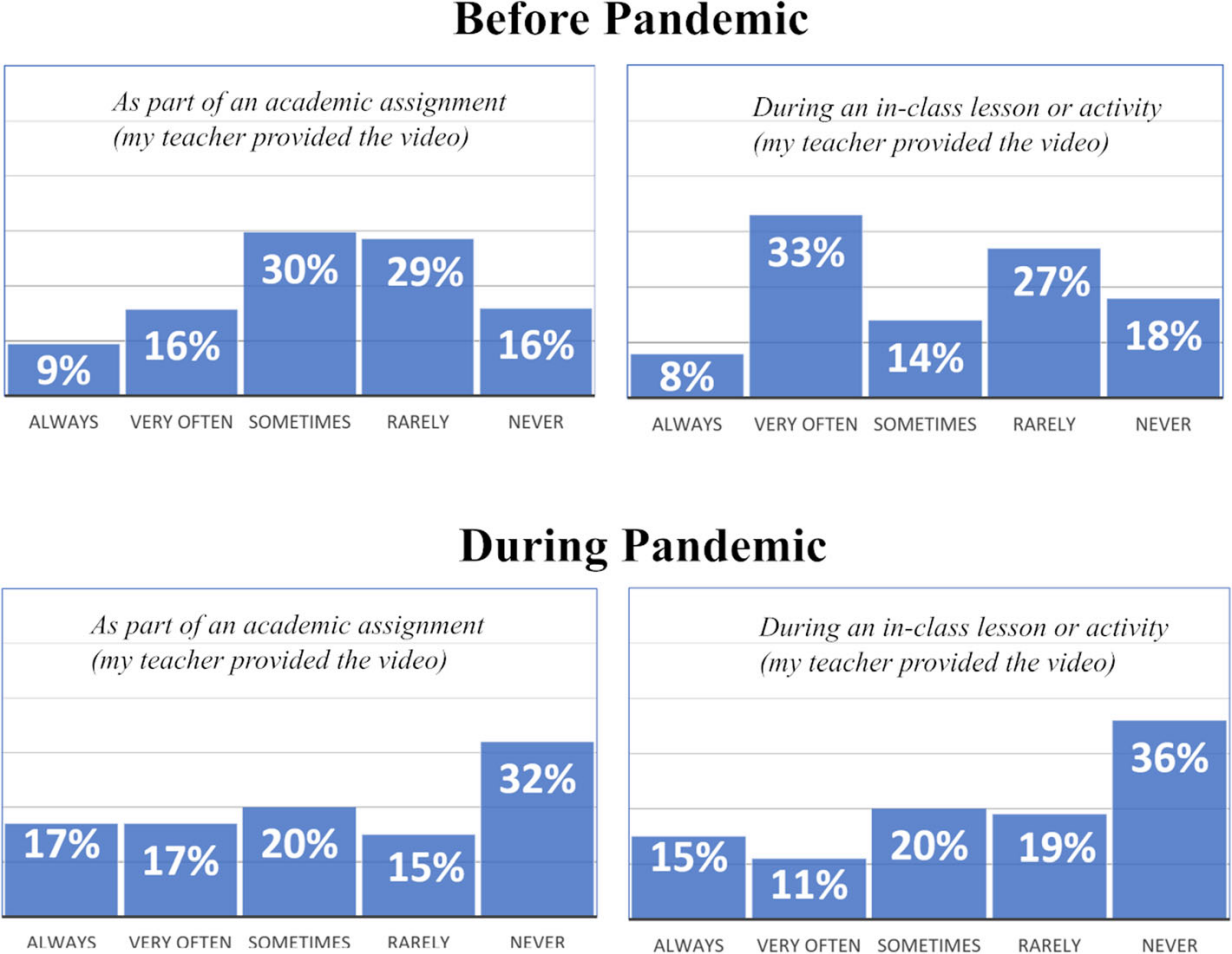
Teacher-driven use of video before and during the pandemic

Figure 9 represents student-reported data for how science teachers use video in instructional contexts are reported.

Data indicates that trends for teacher-directed use of video to accomplish academic tasks before and during the pandemic are in relatively distinct in contrast to trends for student-directed use (presented in Fig. 7). The number of students reporting that their teachers provided videos for academic assignments or activities did appear to increase slightly during the pandemic. However, while students reported that their *own* use of video increased substantially in response to the shift to virtual learning, the number of students reporting that their teachers never provided videos for academic assignments or activities doubled during the pandemic.

We found the comparisons of teacher use of video before and during the pandemic somewhat surprising given that most students were in primarily online learning environments during the pandemic. We expected that online video would be more accessible and, as such, utilized more often. We wondered whether there were other ways that teachers were using online videos to support student achievement during the pandemic; for example, showing a video during an online lecture or providing a list of links in an online discussion forum. We also wondered how teacher use of video for science classes may compare to the use of video in other disciplinary classes during the pandemic. Therefore we added two items to the questionnaire to directly ask about teacher's use of video. We asked:

*Compare before the pandemic to during the pandemic: how often did your teacher use videos to teach science?*

**Fig. 10:**
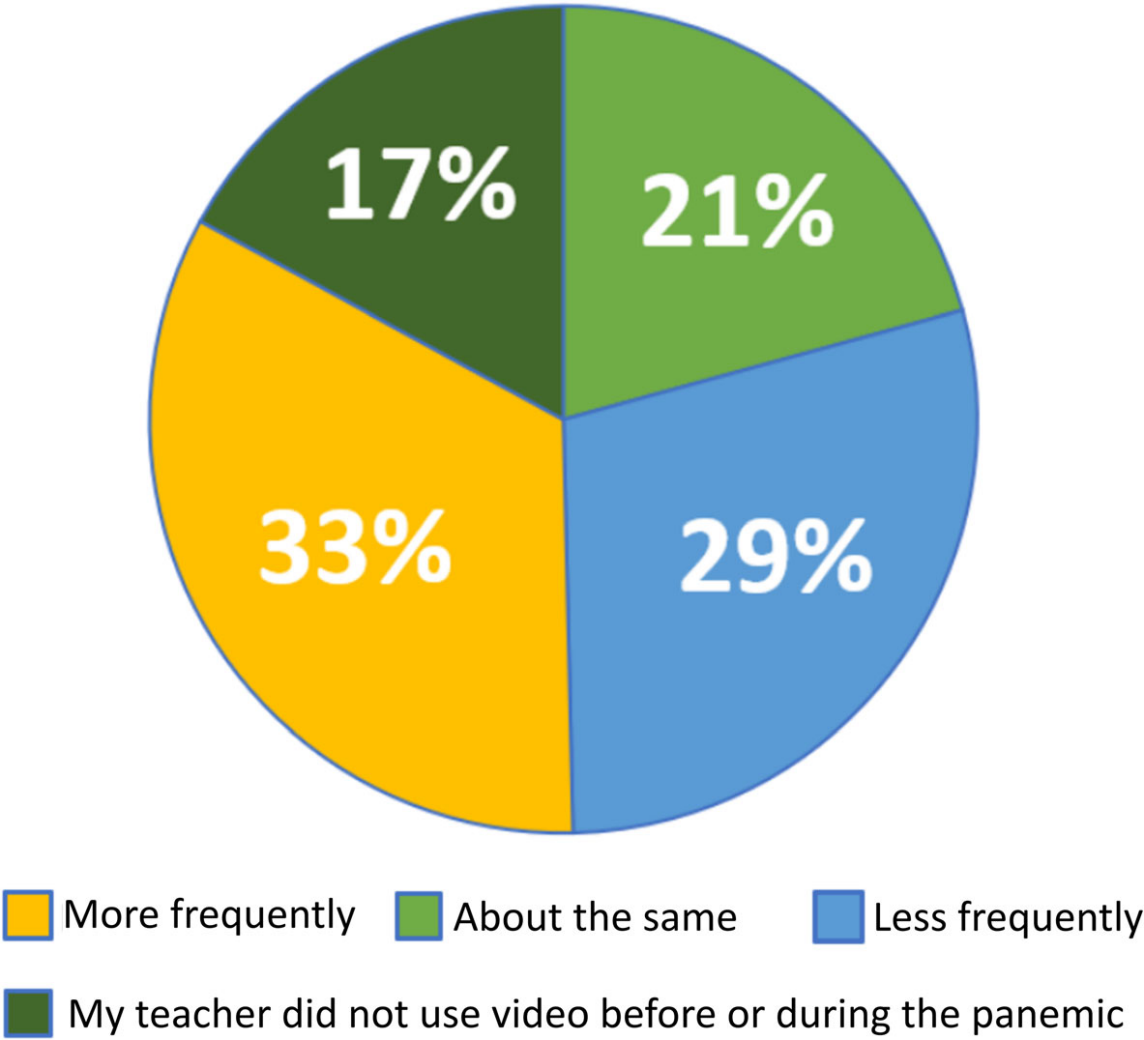
Comparison of teacher use of video before and during pandemic

**Fig. 11:**
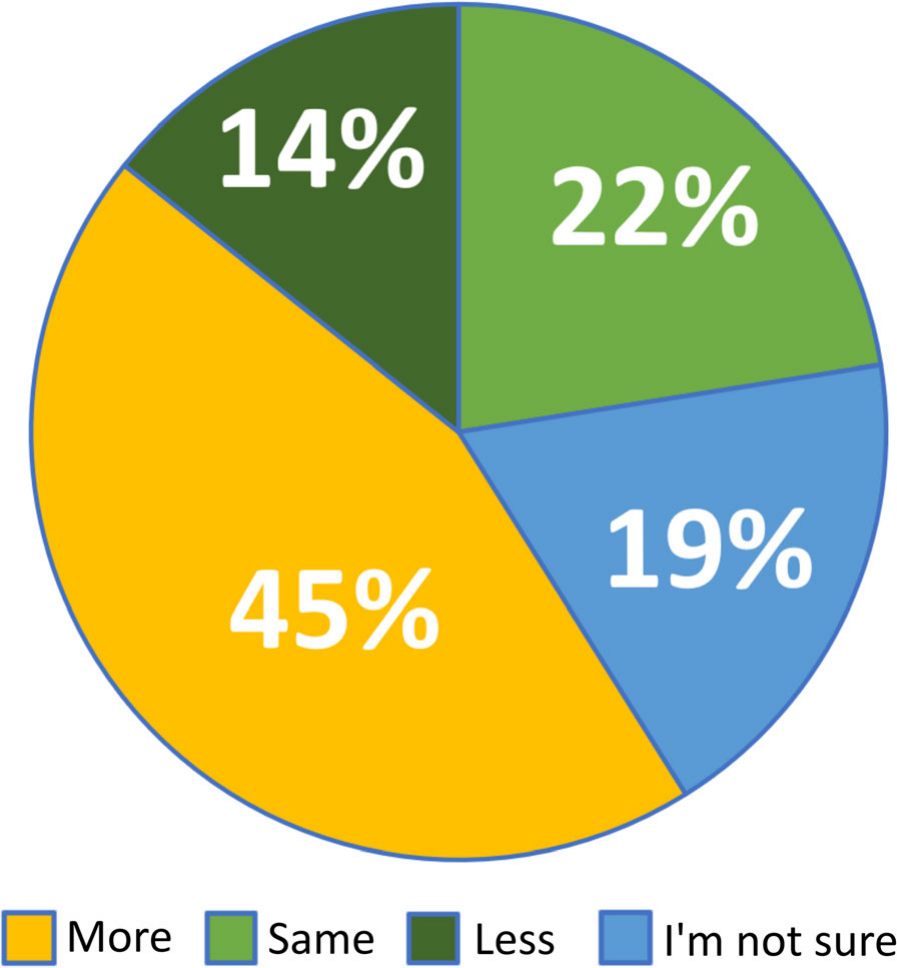
Teacher use of video during pandemic compared to other classes

A total of 540 responses were received and are presented in Figs. 10 and 11.

These data sets provide a clearer overall picture of teachers’ use of video. Figure 10 reports the frequency of teachers’ use of video to support academic activities in science before and during the pandemic. Of note is that 33% of teachers were reported to use video *more frequently for instruction*, which is considerably higher than reported in Fig. 9 for only *assignments* or *in-class activities*. This increase is likely due to student responses including a wider variety of video use by teachers than is represented by data in Fig. 10. This data set does, though, does echo the general trend revealed in Fig. 9 of decreased teacher-driven use of videos during the pandemic with 67% of teachers using video the same amount as before the pandemic, less often, or not at all.

Almost half (45%) of students reported their science teachers used video *more* than other classes during the pandemic and only 14% reported their science teachers using video *less*. It is possible that the 19% reporting “I’m not sure” represents situations where video use was low or non-existent for all of the students’ classes or that they don’t remember; however, it is not possible to tell from this data set.

### Looking forward: the return to in-person learning

Our first two research questions focused on understanding the role that video played within science learning ecosystems before and during the pandemic-driven disruptions to traditional instruction. Our third research question sought to explore students’ preferences for using video in post-pandemic academic contexts given their recent experiences. At the conclusion of the online questionnaire, students were asked:

“*Do you think teachers should use online science instructional videos with their students when classes are taught in-person? Why or why not?*”

Despite being an optional question that included a space for a “long answer text” response, only six of the 1147 survey participants chose not to answer this question and a total of 1141 responses were recorded.

#### Primary coding

Our systemic analysis revealed four primary codes for responses that indicate whether the student answered that teachers should use video for future in-person classes, should use video in certain circumstances, should not use video, or should not use video except in certain circumstances. Approximately 98% (1115) of the total responses were able to be coded with one of the following primary codes:

- *Yes***:** teachers should use online science with their students when classes are taught in-person
- *Yes-Conditionally*: teachers should use online science with their students when classes are taught in-person with conditions
- *No*: teachers should not use online science with their students when classes are taught in person.
- *No-Conditionally:* should use not online science with their students when classes are taught in-person with conditions

A small number of responses (26 responses representing 2% of the total) were unable to be attributed to one of the four primary codes. These responses were determined to be ineligible for further analysis. Table 5 in Appendix A details responses determined to be ineligible for consideration. Table 4 represents the codebook for the primary codes that were inductively developed through our systematic analysis.

**Table 4:** Primary Coding Scheme

Code	Description	Example(s)	Total Responses	Percentage of Total
Yes	Response clearly indicates a yes response without conditions or qualifications	“Absolutely! Learning from another person gives students a different perspective on the subject matter.” “Yes because they are very clear and precise”	936	84%
Yes, Conditionally	Response indicates a yes response but conditions and/or further qualifications are explicitly named or can reasonably be inferred.	“That depends actually. Most of the time yes, but if the teacher is already competent, they can be missed.” “Depends, sometimes the curriculum varies and science videos aren’t able to help. Especially in Australia.”	76	7%
No	Response clearly indicates a no response without conditions or qualifications	“No, if I wanted to watch videos on a subject I would take an online class.” “Nah, would not be as interactive. Students may be uncofmratble [sic] asking questions when not in an open discussion.”	99	9%
No, Conditionally	Response indicates a no response but conditions and/or further qualifications are explicitly named or can reasonably be inferred.	“No. It depends on the class. Sometimes videos don’t cover what the professor wants you to know.” “Not all the time”	4	< 1%
Total Responses eligible for Further Analysis: 1115, 98% of total				

**Fig. 12:**
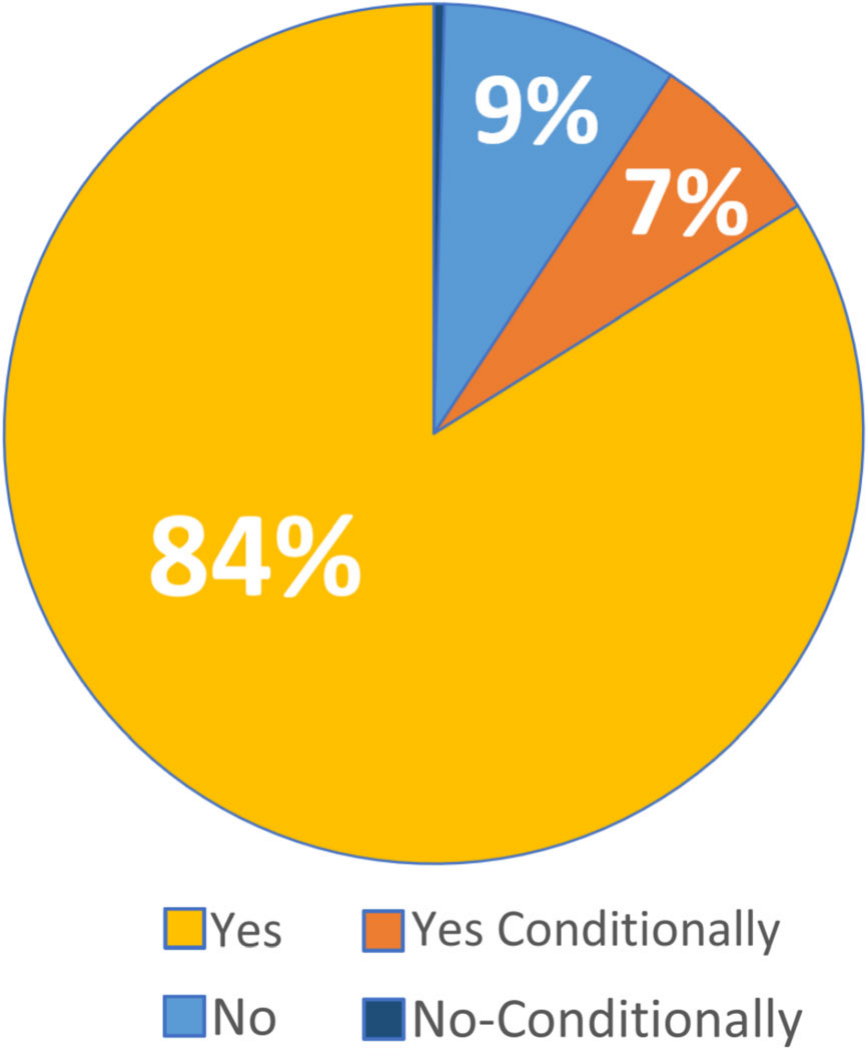
Should teachers use video when classes are taught in person?

The 1115 responses that were categorized into our four primary codes indicate that approximately 91% of these students report that teachers should use online science instructional videos with their students when classes are taught in-person either unequivocally or within certain parameters or conditions. Approximately 9% of these students report that teachers should not use online science instructional videos with their students when classes are taught in-person either unequivocally or only unless certain parameters or conditions are met. These findings, presented in Fig. 12, respond directly to our third research question, *How do students prefer video to be used for science learning post-pandemic*, but they are also notable in the context of our other research questions and indicate students also believe that videos should continue to be used *by teachers* for in-person instructional programs. In other words, the majority of students who responded to this survey would like to see videos continue to be included as legitimate resources for science learning ecosystems post-pandemic.

#### Secondary and tertiary coding

During our analysis, we noted that approximately 85% of the responses to this survey question included long answers that contained enough data to allow us to make sense of the *reasoning* behind the students’ responses and better understand when, why, and how they advocate for teachers to use this resource. Therefore we continued our systematic review and inductively developed additional levels of codes under which the responses could be categorized. The four secondary codes that emerged during further analysis include General or Unspecified Reasoning, Usage, Inherent, Learner.

### General or unspecified reasoning

Responses given this classification are those that indicate whether the student advocates for teachers to use videos, but that do not offer enough detail that allows for further coding. Examples of responses given this code are those that broadly reference whether videos are “helpful” and those that refer to whether or not they are “necessary” (but do not provide detail with which to further refine the classification). Examples of responses coded General/Unspecified include “*Yes, they help so much,”* and “*Yes teachers should use science instructional videos because I guess then the student will be able to understand stuff better.*”

### Usage

This code was assigned to responses that focus on ways that video could or should be used within formal instructional programs. These responses typically emphasize whether videos should be used instead of or in *addition* to other, more traditional resources to supplement and/or reinforce concepts. For example, “*Yes it’ll reinforce ideas taught in the classroom;*” and “*They would definitely be useful for practice problems or even kids who struggle with topics and need the extra help.”*

Some of these responses note that videos should *only* be used in a supplemental capacity to address gaps in student understanding. For example, one response given this code reads, *“No; if the teacher is confident enough to teach their own class the content they shouldn’t be showing other videos, only refer to them for further understanding of a particular concept or help with homework; or if they want to cement a point/provide examples.”* The Usage code was also applied to responses that indicate the student does not believe videos to be appropriate for use in class but do think they are appropriate for students to use on their own.

### Inherent

Responses labeled with the Inherent secondary code are those that indicate reasoning related to characteristics that are unique and/or essential to video itself. In other words, the response indicated that the student was attributing the reason video should or should not be used by teachers to distinguish features that are inherent to online videos and/or the platform through which they are shared.

### Learner

The secondary code category, Learner, was assigned to responses that emphasized the impact that teachers’ use of online videos has on the learner and learning.

**Fig. 13:**
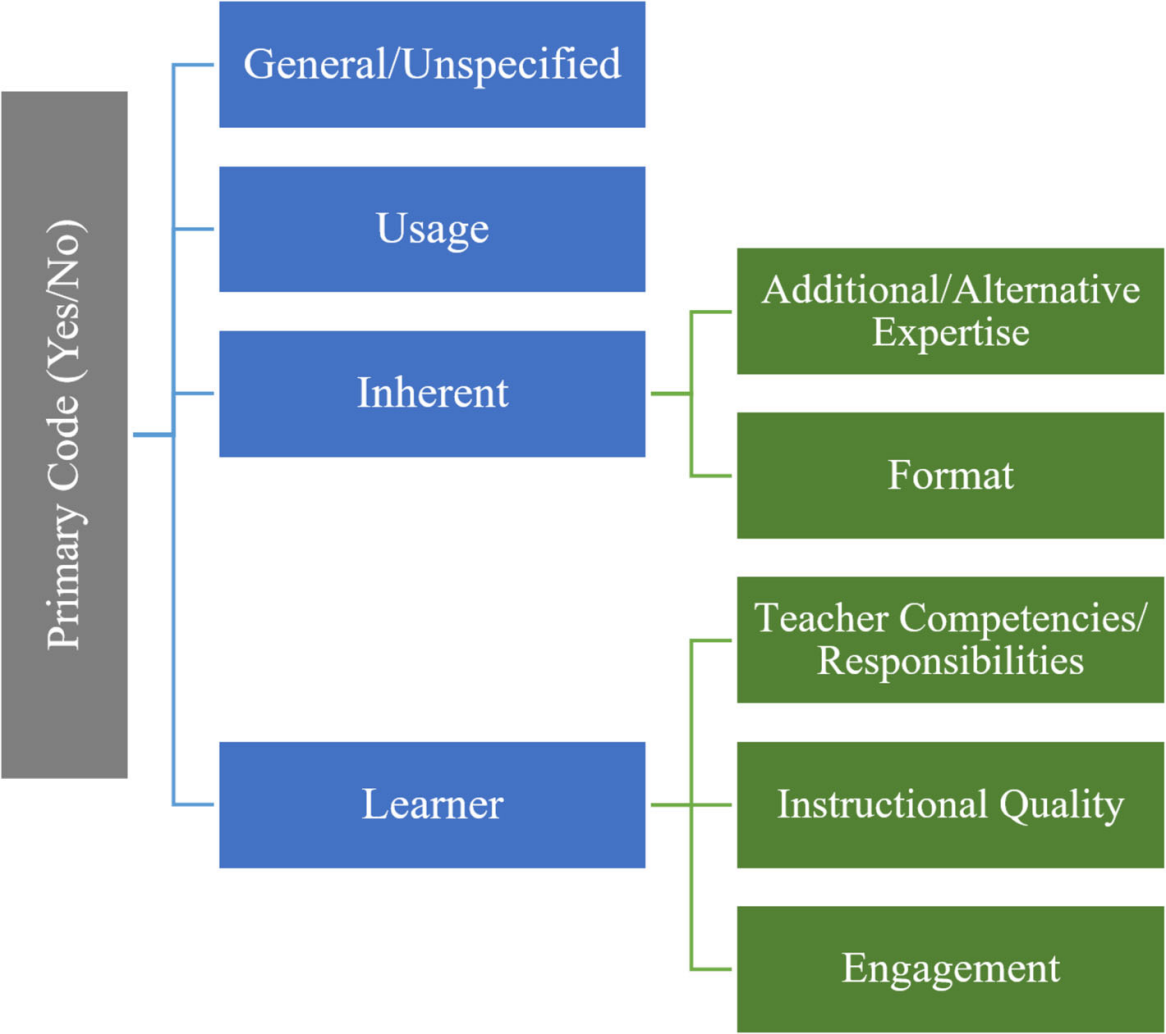
Primary, secondary, and tertiary codes

Secondary codes, along with tertiary codes, are presented visually in Fig. 13. In addition, the codebook for secondary and tertiary codes is available in Table 6 in Appendix B.

### Findings from secondary and tertiary coding

**Fig. 14:**
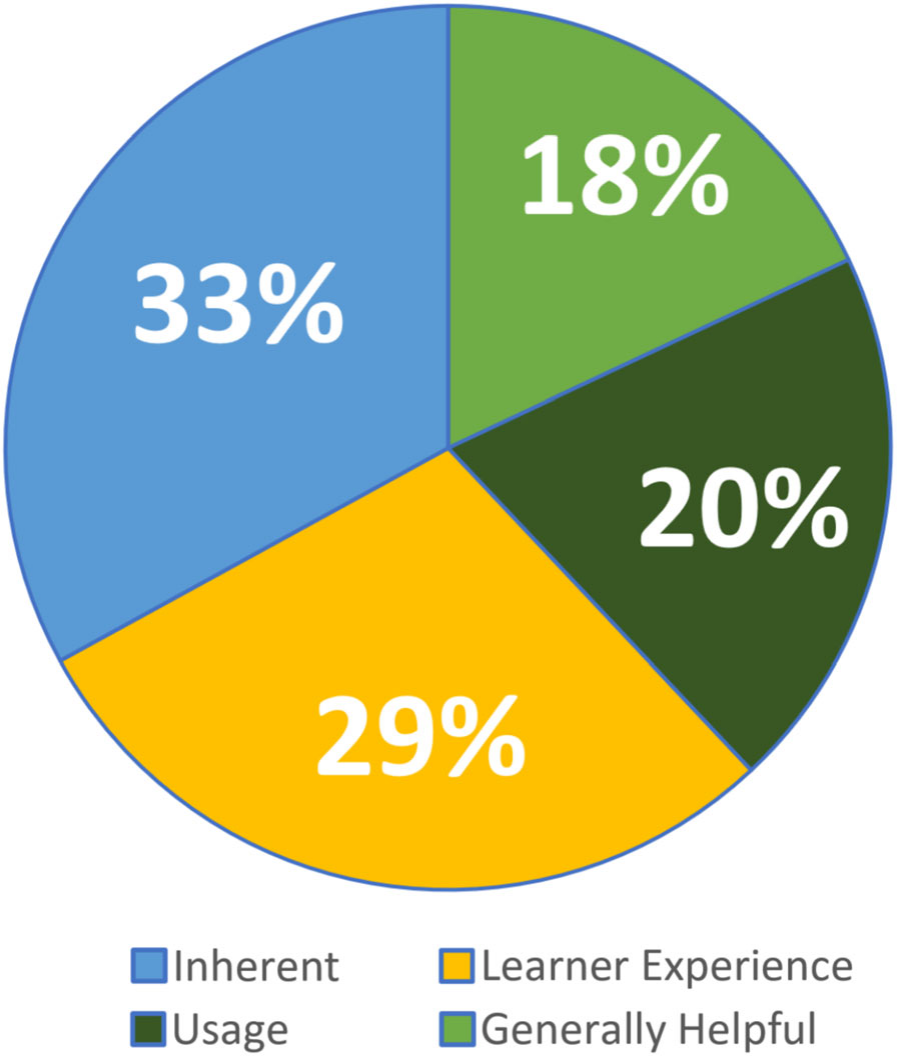
Reasoning for why teachers should use video for in-person learning

Secondary coding provides insight into why students believed or did not believe that video should be used by teachers. Examining the responses under the Yes and Yes-conditionally primary categories, we found that 33% of the responses focused on qualities Inherent to the video, 29% pointed to the positive impacts of video on the Learner and learner experiences, 20% emphasized the advantages of video Usage, and 18% of the responses were General or Unspecified (Fig. 14).

Our attention was drawn to the idea that the majority of responses focused on the affordances that are attributed to features unique and inherent to online video. This suggests that students believe that the format, structure, or inherent *nature* of video as a medium for supporting learning is an advantage. We were also drawn to the large number of responses that described the impact that video has on the learner experience. As shown in Fig. 13 above, our analysis of these two secondary coding categories (Inherent and Learner) indicated more nuanced inferential patterns within and across responses labeled with these codes, and thus further analysis was conducted. Both of these two codes were subsequently broken down into tertiary categories. Tertiary labels for responses under the Inherent category include:

- *Additional/Alternative Expertise*: Applied to responses in which the reasoning prioritizes affordances or drawbacks related to the idea that videos offer a different perspective, style of delivery, or variation of information than other resources (such as teacher lecture). For example, the following response was coded with the primary code of Yes as well as Inherent for a secondary code and Additional/Alternative Expertise as the tertiary: “*Yes, it’s beneficial to have information taught in a different way by a different teacher. Some students can learn better with a different style of teaching.*
- *Format:* Applied to responses that prioritize the affordances or drawbacks associated with the format of videos and video-sharing platforms. This includes references to the organization of video content, the length of videos, visual elements that are inherently possible with videos (such as animations and simulations), the ability of the user to control the instructional delivery (in other words, users can rewind, pause, replay), as well as references to the online medium through which videos are shared. For example, “*YES! the student can pause as many times as needed, and take more thorough notes*.” The Inherent, Format codes were also applied to responses that highlighted the advantages of using video to support classroom learning due to students’ familiarity and preferences for using technology and/or online video sharing platforms. For example, “*Totally! I believe that this kind of teaching is much more effective to students than the one without videos and that’s because kids-teenagers-college students today, find anything related to technology and exposure really intriguing and tempting.*”

**Fig. 15:**
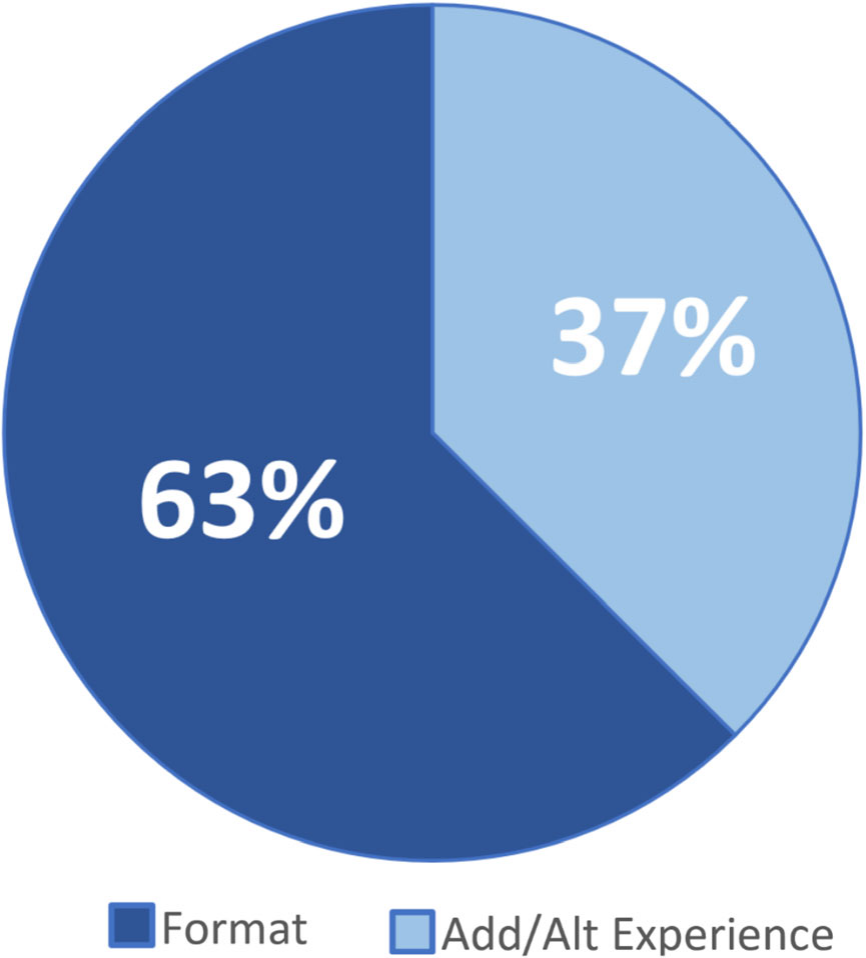
Tertiary breakdown of reasoning inherent to video

Further analysis of the responses coded Inherent indicates that 63% emphasize the affordances of the online video resource Format and 37% focus on advantages related to the fact that the video offers an Alternative and/or Additional Expert or teacher as a source of information and qualities specific and unique to the video platform itself. Figure 15 represents the tertiary breakdown of the Inherent category.

The secondary code category, Learner, was also subdivided into three tertiary codes.

- *Teacher Competencies/Responsibilities:* Applied to responses in which the reasoning specifically prioritizes the influence and/or impact of video use on the content knowledge, teaching style, pedagogical choices, and/or effort of the instructor --whether referring to the classroom teacher or video content provider. For example, the following response was coded with the primary code, NO, and subsequently labeled with the Learner, Teacher Competencies/Responsibilities: “*Teachers should teach what they know. What is the job of a teacher if he/she just plays videos during class.*” Some responses that were primary-coded Yes (teachers should use video for in-person instruction) were also assigned to the Learner, Teacher Competencies/Responsibilities category. For example, “*Yes, if students find that teachers are incapable of explaining concepts, DR B is a great alternative to try and learn the material.”*
- *Instructional Quality:* Applied to responses for which the reasoning prioritizes the quality of instruction delivered by the video content provider and/or video. These responses indicate that videos are highly effective, more effective, or less effective at supporting student understanding. For example, the response, “*Yes, they are very easy to understand and the information that is given is clear*” was given the Instructional Quality tertiary code. Note that responses given this code do *not* attribute the impact on learning to characteristics of the video platform itself and are thereby distinguished from responses assigned to the Inherent, Format category.

**Fig. 16:**
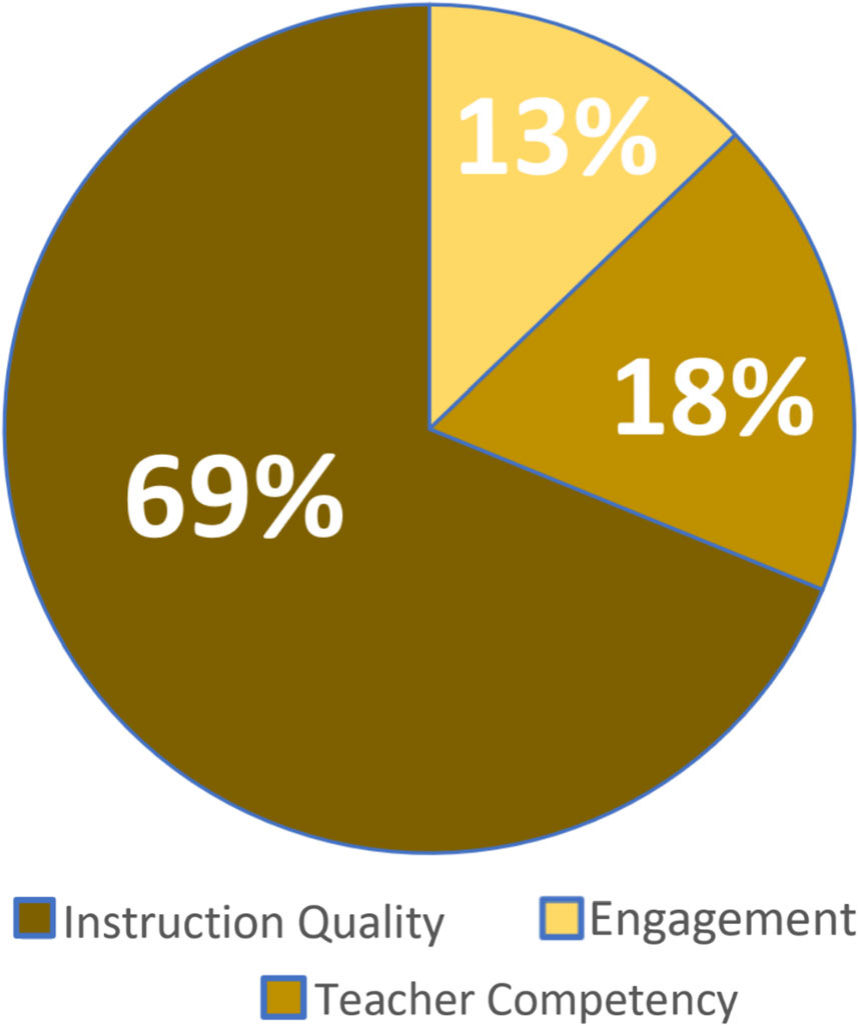
Tertiary breakdown of reasoning for learner experience

Further analysis of the responses coded Learner indicates that the majority of responses (69%) focused on the advantages of video for supporting the learner with an emphasis on the quality of instruction that is made available through videos (particularly when compared to what is offered in traditional, lecture, and/or textbook-based instruction). Responses that indicated videos should be used to support learning and that specifically point to the relative competencies of the classroom teacher and online video instructor represented 18% of these responses (Teacher Competency). The value that video adds to the learner experience by increasing engagement with the content or topic, Engagement, was attributed to 13% of responses in the Learner category. Figure 16 represents the tertiary breakdown of codes under the Learner category.

### Member-check

The YouTube platform provides functionality to conduct polls of subscribers and other visitors to a YouTube channel. These polls are posted to the Community Tab in the same manner as recruitment messages posted earlier in the study. When a poll is posted to the Community Tab, subscribers are automatically notified, and they can follow a link to the poll if interested. After taking the poll, subscribers can see how others have responded but are not able to retake the poll.

Polls are best seen as a way to member-check findings with participants and a wider audience and receive feedback in the form of comments. Polls are limited in that no information, demographic or otherwise, is available to the researcher as they are anonymous. It is anticipated that the individuals participating in the poll are viewers of the first author’s channel, and their demographics mirror those of the channel. However, this is not possible to confirm.

After analysis of questionnaire data, subscribers to the first author’s YouTube channel were asked to complete an online poll about our findings. They were provided with a brief explanation of major findings as well as a link to a longer, two-page summary that included Figs. 7, 8, and 9. A link to the summary was posted to the Community Tab of the first author’s YouTube Channel. Participants, as well as subscribers to the channel, were asked to view the findings and respond in the Comments section of the Community Tab or via email.

Subscribers to the channel were asked to look at the findings, as well as a two-page document providing graphs and charts with explanations, and complete a poll with their level of agreement/disagreement. The following is the text of the post to the Community Tab:

**Table:** 

Here are the top three findings from our recent research. Please take the poll and leave your thoughts in the comments! You can see a short report with initial findings at [web link]. • Students’ personal use of video for learning science increased substantially during the pandemic. • The majority of students reported that their science teachers’ use of video for instruction was the same or less during the pandemic. • Post-pandemic, students plan to continue using science videos for learning and want teachers to do the same. Do you agree with our research findings?	

Of the 2000 individuals completing the poll, 86% *agreed* or *mostly agreed* with our findings while 6% *mostly disagreed* or *disagreed*. A total of 8% responded as *neutral*. A small number of written comments were received, with three mostly agreeing or slightly disagreeing. For example:

*I mostly agree with the research, except that, as previously mentioned by others, I had teachers who would never even assign videos, but now use them in essentially every class. I had no habit of studying for school with online videos before the pandemic whatsoever, I would only at times watch science related content on YouTube for leisure. As it has proved to work well with me, I do not intend to stop using YouTube or your channel as sources to guide me through school and learning experiences in the future.*

## Discussion

In this study, we found that the Covid-19 pandemic brought about a clear shift in students’ use of resources in the science education learning ecosystem. Both questionnaire data, and data on video views for the first author’s YouTube channel from 2018 to the present point to the rapid growth and sustained use of instructional science videos. Moving beyond documenting changes in student use before, during, and while emerging from the pandemic, a larger question is what characteristics are inherent to video that led to this change. What factors influenced the changes in students’ choices and preferences for video to support their science learning during the pandemic? Further, will these changes continue to influence the science education learning ecosystem when we shift to post-pandemic contexts?

### Students’ use of video

Our first research question asked, “*How has student use of instructional science video changed during the Covid-19 pandemic?”* We found that, as students shifted to online and hybrid contexts as a result of the pandemic, they relied more on videos to understand science ideas and information and for support with homework and exam preparation. Video quickly became a central part of their academic learning efforts.

Video, as a learning resource, does not exist in isolation and it is productive to consider how students use video in the context of the larger learning ecosystem of resources. A comparison of three resources: lecture, textbook, and video provides insight into changes observed during the pandemic.

In the context of *in-person* learning, students rank videos after lectures, labs, and textbooks as the most effective learning resource. In *online and hybrid* contexts, video replaces lecture as the most effective. To understand this change it is necessary to look at what students find compelling about instructional science videos in online and hybrid learning contexts. In other words, what does video offer that is missing from more commonly used resources like lectures and textbooks?

### Students’ preferences for future use of video

As the fall 2021 semester draws to a close, many countries continue to battle the effects of the COVID-19 pandemic. School systems are still working to recover, returning to in-person instruction after more than a year of learning in virtual environments that forced students and teachers to adapt to using technology and resources in new and innovative ways. This research has indicated that students viewed online instructional video as a favored resource for learning science before the pandemic and utilized video even more during the shift to online learning. A notable question, however, is whether the increased use will be sustained as students return to primarily in-person learning.

We felt reasonably comfortable, based on the first authors’ YouTube channel analytics on the number of video views from January 2018 through October 2021, that the increased use of videos will continue. However, we sought to understand the potential for video to play more formal roles in post-pandemic learning ecosystems. This would be at least partially dependent on *teachers’* willingness to use and/or promote videos for instructional purposes. This study allowed us to collect data on students’ perspectives on whether and how the increased use of online video that was recorded during the pandemic might be sustained as they return to classrooms. We asked, “*How do students prefer video to be used for science learning post-pandemic?”*

The data strongly indicate that this sample of students believes that online videos should be used *by teachers* for in-person instructional programs in the future. Student rationale focused mainly on the inherent qualities of video as a technologically based medium, the positive impact of video on the learner and learning process, and the general, unspecified helpfulness of video. Several responses advocating for teachers to use videos center on the ability of videos to leverage animations, simulations, and other elements that allow students to visualize concepts and mechanisms in meaningful ways.

Students also point to the affordances videos provide for allowing the learner to have increased individual agency over the ways that instruction was delivered to them. For example, students can pause and rewind videos to replay, review, and reflect on ideas as needed. As one student noted, “*Videos are more convenient for students so when we think the teacher is going too fast we can rewind the video or pause it to have time to think*.” Another response appreciates the ability to pause a video in specific comparison to direct instruction from classroom teachers: *“Being able to pause a video and write down everything important is amazing and something we don’t get to do in a person lecture.”*

Responses emphasizing inherent advantages that video provides also point to the value in simply having an additional or alternative source of instruction. For example, “*Yes, another point of view on teaching is never a bad thing, it will most likely aid the students in their learning;”* and “*Yes, because students will understand a particular concept from 2 different teachers (school teacher and the teacher in video) making high chances that they clarify their understanding.”*

Interestingly, several responses also advocate for the use of video because of the ubiquity of technology in everyday life and that online resources represent a medium with which young people are highly familiar. For example, *“Yes, because nowadays that’s the format of our entertainment and making our education in the same format will make it more fun and easier to learn.”*

Students who explained their reasoning for believing that teachers should use video for in-person learning also emphasized the value that using video has on the learner and the learning process (29% of positive responses). Many of these responses point to the quality of the instruction available through online videos, most of which are produced by experts in science and education, the ability for videos to compensate for gaps in teacher knowledge and/or pedagogical efficacy, and the engagement and motivation for learning that videos inspire.

### Teachers’ use of video during the pandemic

We also investigated the research question: “*How has teacher use of instructional science video changed during the Covid-19 pandemic?”* Understanding teachers’ use of instructional science video is more challenging with the current dataset, which relies on student self-report data. However, data suggest that the majority of teachers used video the same or less during the pandemic. Of note is that 17% of teachers didn’t use video before or during the pandemic, similar to findings by Pattier (2021) for secondary and university settings. For some teachers, video is not seen as a useful or accessible tool to support student learning.

Looking at video use during the pandemic from the perspective of student-directed (Fig. 8) and teacher-directed use (Fig. 10), most teachers did not increase their use of instructional science videos as a means to support student learning. Only 33% of students reported their teachers used video more during the pandemic.

It is not clear why many teachers did not increase their use of instructional science videos to support student learning during the pandemic given the extended shift to an online and hybrid learning environment. As the shift online was abrupt and challenging for many teachers, it may be that teachers did not have the time or resources to include video into their lessons. It may also be that the factors driving student use are not perceived as valuable to teachers. For example, students’ preference for choosing from multiple perspectives and presentation styles in learning a topic may not resonate with teachers.

There may also be cultural and age-related beliefs about learning and the role of teachers in science education. Further research into teachers’ beliefs about the role of video in science education, and in the science learning ecosystem, is needed. In addition, the majority of YouTube instructional science videos are created by current and former teachers. Understanding their goals, motivations, process for selecting topics and creating videos, could provide insight into teacher use of video.

## Conclusion

Our observations of the sustained elevation of views of science video during periods of online learning inspired us to explore the role that video plays within the constellations of resources and contexts of science learning ecosystems. Our reviews of the literature indicated that, generally, not much is known about the roles that YouTube videos play for students and teachers. The pandemic-driven disruptions to the stability of learning ecosystems have, in our view, heightened attention on the educational value of online videos and by studying changes in how students used videos in the relatively early days of the pandemic, we might better understand the role that video play in educational ecosystems moving forward.

For participants in this study, online instructional science video became a core learning resource during the pandemic. However, video is just one part of a larger science learning ecosystem made up of formal and informal learning resources and experiences. All these resources contribute and interact to provide students with science experiences and ideas. To be clear, we are not advocating that video should become the primary medium for science education or making claims about quality or effectiveness. It does seem, however, that video is a resource that is perhaps undervalued by teachers, curriculum developers, and other academic decision-makers relative to students’ habits and preferences. Better understanding the ways students use videos could be valuable for informing student-centered curriculum and pedagogy moving forward.

As we began our research, we envisioned the world would soon emerge from the pandemic with vaccines and new therapeutics. Approaching two years with Covid, this does not appear to be the case and we may be entering what some are calling the Covid Era (Kolata, 2021). In many countries, students are going back to school but the context of school has changed as has students’ use of online video for learning science.

The use of instructional science video on platforms like YouTube is a nascent field of research. Unlike the mature and extensive research literature on science textbooks, there is little research available for video on platforms like YouTube. Further, online video is complicated by multiple creators with differing skills and motivations, a strong social component, issues of access to reliable internet, and a more visible commercial connection to the companies hosting the platforms. New tools for data collection and analysis are necessary to address this complexity. There is a need for a research agenda to guide work in this emerging area. Based on our recent work and experiences with the YouTube platform, we suggest several core areas to move the field forward.

One area in which further investigation would be valuable is how students use online instructional video relative to other resources within and across the larger science learning ecosystem. This would be of value as the sample in our study only represented students subscribed to a chemistry-focused YouTube channel. Variations in epistemological and pedagogical norms across academic science disciplines may influence the efficacy of the types of videos that are leveraged in support of learning as well as how they are used.

Investigation into the factors that influence teacher use of video is also a valuable area for further study. Our research indicates that, from the student perspective, there is a disconnect between the ways that students and teachers use videos. A deeper understanding of the motivations for whether and why teachers choose to use videos within their instructional programs is critical for the construction of science learning ecosystems.

The creation of instructional science videos also needs to be carefully explored. Who are the individuals creating this widely used content? What are their goals, motivations, and processes for selecting and creating? It appears that many, if not the majority, are practicing or former teachers. How does this fit in with their classrooms, their professional development, and student perceptions of instructional science videos?

Finally, compared to the use of textbooks and other elements of the science learning ecosystem, online science videos are relatively new to the science education domain. While there are challenges in studying video use, videos also provide a window into what students choose to learn from when able. What does it mean for science education when students have ready access to content from a multitude of delivery styles and perspectives? How can this be effectively employed to provide more time for teachers to engage in other high-value activities such as laboratory experiences, problem-solving, and problem-based learning?

## Limitations

We acknowledge that many students do not have access to technology and could not take part in this study. As such, care must be taken in generalizing findings to all students. Further, participants were from 83 different countries as well as different academic levels, from middle school to graduate school. For this article, it was not feasible to disaggregate across all groups.

Participants in the study were subscribers to a particular YouTube channel about chemistry, and while similar to other science education YouTube channels, differences likely exist. Further, while this is not the only source of videos for their learning, students may feel an emotional connection to the first author. This could potentially influence them to report a more favorable view of online videos. At the same time, they are motivated and likely to take the completion of the questionnaire seriously.

Due to the global nature of our data collection, as well as challenges presented by the pandemic, we were not able to definitively validate the age or educational level of participants. Data from YouTube analytics does show that US viewership closely follows the school calendar with large decreases over holidays and breaks, suggesting that viewers are students. This is further supported by data on viewer age provided by YouTube analytics which places 78.5% of viewers under 34 years of age, with the majority of these under 24 years of age. The nature and content of comments on videos across the first author’s YouTube channel support the assertion that students are the primary audience. Therefore, while we are not able to definitively confirm the ages and education levels, we believe that self-report data from participants on age and education level is accurate.

A further limitation is that questionnaire data for this study were self-reported by participants. For demographic data, students tend to be truthful (Blinka & Smahel, 2009). However, the accuracy of self-report data by students on how their teachers is less certain. Our findings do mirror published research on teachers’ use of video (Pattier, 2021) but further research is needed on teachers’ use of video.

A core component of our research is in documenting changes in the use of video during the pandemic. We are confident that the self-report on usage is accurate as YouTube analytics data mirrors the increase in viewership found in self-report data. Further, we compared data with a YouTube channel of similar size over the date ranges presented in Figs. 2, 3, & 4 and found the same patterns of views before and during the pandemic (M. Maribel, personal communication, October 25, 2021).

## Data Availability

Datasets are available from the corresponding author on reasonable request.

## Appendix A

**Table 5:** Primary Coding Scheme: Coded Responses Ineligible for Primary Codes

Inductive Code	Description	Examples	Number
IDK – I Don’t Know	Response clearly indicates the participant does not have a clear response to the question.	“I’m not sure” “i dont know. Better watch Dr. B videos”	7
M – Maybe	Response indicates indecision and more specific coding would require undue inference.	“Maybe. The time lost to AV issues, is a real concern. It can be distracting and take you out of focus on the lecture. I can’t tell you how many minutes of a valuable lecture of lost to bad AV issues. When lecturers embed the videos in their slides, its easier because they can just play it directly from their presentation.” “Maybe”	2
NR – Non-Responsive	Response does not clearly respond to the question.	“To be completely honest my teacher sent me the link of the video and that’s why i am filling this form to help you people with your research (P.S ALL THE BEST WITH THE RESEARCH)” “They can but they wont. They always pick some crappy videos that are not so interesting. But I really wish that some of the phenomenal videos need to be used during teaching (like dr. B < 3 < 3 < 3)”	4
UC – Uncodable	Response is unclear, irrelevant, and/or excessive inference is required to assign a coding pattern to the response.	“I would recommend this to my classmates and teacher but since it’s in english and it’s not our native speaker so it could be lit bit hard to understand.” “Good.”	13
Total Responses Ineligible for further analysis: 26.2% of total			

## Appendix B

**Table 6:** Codebook for Secondary and Tertiary Themes

Primary Code	Secondary	Tertiary
• YES • YES-CONDITIONALLY • NO • NO- CONDITIONALLY	**INHERENT** Reasoning is related to characteristics that are unique and/or essential to video.	ADDITIONAL/ALTERNATIVE EXPERTISE Reasoning prioritizes the affordances or drawbacks of the idea that videos offer a different perspective, style of delivery, variation of information, etc.
		FORMAT Reasoning prioritizes the affordances or drawbacks associated with the format of videos and video-sharing platforms. This includes: • organization • length • medium (online) • visual elements made possible via videos (simulations, animations, etc.) • User-control (can rewind, pause, fast-forward)
	**LEARNER** /LEARNING Reasoning is related to the impact science instructional videos with their students when classes are taught in-person will have on learners and learning	TEACHER COMPETENCIES/RESPONSIBILITIES Reasoning specifically prioritizes the influence and/or impact of video-use on the content knowledge of the instructor (in-person teacher or video content provider), teaching style, pedagogical choices, etc.
		INSTRUCTIONAL QUALITY Reasoning prioritizes the influence and/or impact of video-use on teaching and learning, generally. For example, the respondent notes that videos are highly effective, more effective, or less effective at supporting student understanding.
		ENGAGE - reasoning prioritizes the potential for video to motivate, engage, entertain, provide an alternative to traditional styles of information delivery.
	**USAGE** The response focuses on ways that video can/cannot be used. For example, in supplemental capacities and/or to reinforce concepts.	
	**GENERAL/UNSPECIFIED** Response notes that video is/is not useful, appropriate, or necessary but does not provide enough details to allow further inference.	
